# Dipyridamole stress myocardial perfusion by computed tomography in patients with left bundle branch block

**DOI:** 10.5935/abc.20150117

**Published:** 2015-12

**Authors:** Estêvan Vieira Cabeda, Andréa Maria Gomes Falcão, José Soares Jr., Carlos Eduardo Rochitte, César Higa Nomura, Luiz Francisco Rodrigues Ávila, José Rodrigues Parga

**Affiliations:** 1 Departamento de Tomografia e Ressonância Cardiovascular, Instituto do Coração (InCor) - Universidade de São Paulo (USP), São Paulo, SP - Brazil; 2 Departamento de Medicina Nuclear - Instituto do Coração (InCor) - Universidade de São Paulo (USP), São Paulo, SP - Brazil

**Keywords:** Bundle-Branch Block, Myocardial Perfusion Imaging, Multidetector Computed Tomography, Coronary Angiography, Dipyridamole, Coronary Artery Disease

## Abstract

**Background:**

Functional tests have limited accuracy for identifying myocardial ischemia in patients with left bundle branch block (LBBB).

**Objective:**

To assess the diagnostic accuracy of dipyridamole-stress myocardial computed tomography perfusion (CTP) by 320-detector CT in patients with LBBB using invasive quantitative coronary angiography (QCA) (stenosis ≥ 70%) as reference; to investigate the advantage of adding CTP to coronary computed tomography angiography (CTA) and compare the results with those of single photon emission computed tomography (SPECT) myocardial perfusion scintigraphy.

**Methods:**

Thirty patients with LBBB who had undergone SPECT for the investigation of coronary artery disease were referred for stress tomography. Independent examiners performed per-patient and per-coronary territory assessments. All patients gave written informed consent to participate in the study that was approved by the institution’s ethics committee.

**Results:**

The patients’ mean age was 62 ± 10 years. The mean dose of radiation for the tomography protocol was 9.3 ± 4.6 mSv. With regard to CTP, the per-patient values for sensitivity, specificity, positive and negative predictive values, and accuracy were 86%, 81%, 80%, 87%, and 83%, respectively (p = 0.001). The per-territory values were 63%, 86%, 65%, 84%, and 79%, respectively (p < 0.001). In both analyses, the addition of CTP to CTA achieved higher diagnostic accuracy for detecting myocardial ischemia than SPECT (p < 0.001).

**Conclusion:**

The use of the stress tomography protocol is feasible and has good diagnostic accuracy for assessing myocardial ischemia in patients with LBBB.

## Introduction

The relationship between left bundle branch block (LBBB) and coronary artery disease (CAD) has been demonstrated in numerous studies, which show LBBB to be associated with an increased risk of cardiovascular mortality^1,2^.

Identification of myocardial ischemia in patients with LBBB is important for risk stratification and clinical management^3^. However, LBBB is an obstacle to the diagnosis of myocardial ischemia due to changes in ventricular repolarization (ST-T segment) in the electrocardiogram (ECG)^4^. Investigation of myocardial ischemia in such patients remains a diagnostic challenge because most functional tests (particularly, myocardial perfusion imaging by scintigraphy) have limited accuracy^4^.

Studies have revealed that LBBB can be associated with fixed perfusion defects when assessed by nuclear imaging despite normal corresponding coronary angiograms. These are most common in the septal area and can be found even when the patient has had a normal coronary angiogram. The underlying pathophysiological mechanisms remain unclear.

Coronary computed tomography angiography (CTA) is an effective and non-invasive method, which is used to detect and characterize coronary lesions. CTA has a high sensitivity and negative predictive value, shown in studies using cineangiocoronariography as the gold standard^5^. The latest generation of CT scanners has made image acquisition possible within a single heartbeat, resulting in images with high accuracy for the diagnosis of CAD with substantial drop from exposure to ionizing radiation^6^. Recently, studies using pharmacologic stress myocardial CT perfusion (CTP) have been reported^7-12^. These give functional information about coronary stenosis. Their accuracy is comparable to myocardial perfusion single photon emission computed tomography (SPECT) and cardiac magnetic resonance^9-11^.

CTP uses first-pass perfusion to assess myocardial perfusion, which is an entirely different mechanism than that used by SPECT. It is unknown if the presence of LBBB would influence the accuracy of CTP. To the best of our knowledge, CTP has never been tested in a controlled manner in a specific group of patients with LBBB.

Our aims were as follows to evaluate the diagnostic accuracy of CTP using a 320-row detector CT scanner in patients with LBBB who were under evaluation for CAD and to compare CTP with SPECT for the detection of myocardial ischemia using quantitative invasive coronary angiography (QCA) as the gold standard. We also calculated the additional value of CTP over and above CTA alone in the diagnosis of significant stenosis (≥ 70% on QCA).

## Methods

A prospective study was conducted in a consecutive patient cohort with documented LBBB. The patients were seen in the outpatient department of our institution and had been referred for the evaluation of CAD with a pharmacological stress SPECT exam (adenosine or dipyridamole).

All patients who agreed to undergo a dipyridamole myocardial perfusion stress CT (Aquilion ONE 320 CT scanner, Toshiba Medical System, Ottawara, Japan) and who had no contraindications were selected. Informed consent was obtained from all participants included in the study.

The exclusion criteria was as follows: contraindications to contrast iodine (such as creatinine > 1.5 mg/dL or known allergy to the contrast); contraindications to the use of metoprolol (such as severe bradycardia < 40 beats/min, second and third degree atrioventricular block, severe aortic stenosis, asthma, chronic obstructive pulmonary disease, and atrial fibrillation); a history of cardiac surgery; previous coronary angioplasty or documented prior myocardial infarction; heart valve prosthesis and other cardiac devices; class III and IV (NYHA) heart failure; pregnancy; BMI > 40 kg/m^2^; and age < 35 years. Patients who refused to sign the consent forms were also excluded.

**Figure 1:**
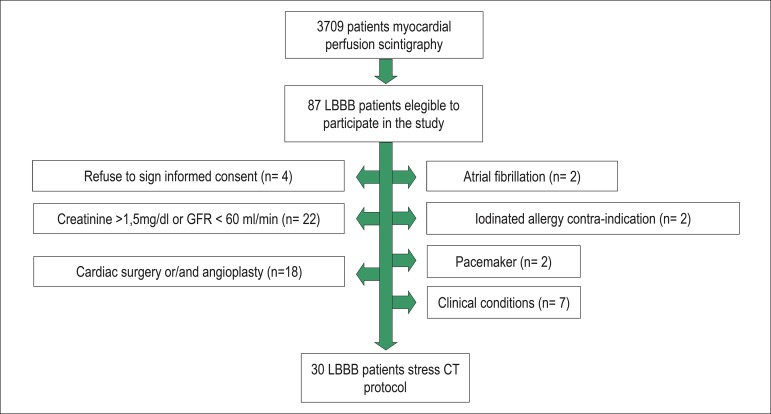
Workflow of patient selection. LBBB: Left bundle branch block; GFR: Glomerular filtration rate; Clinical condition: Heart failure, chronic obstructive pulmonary disease and asthma.

**Figure 2:**
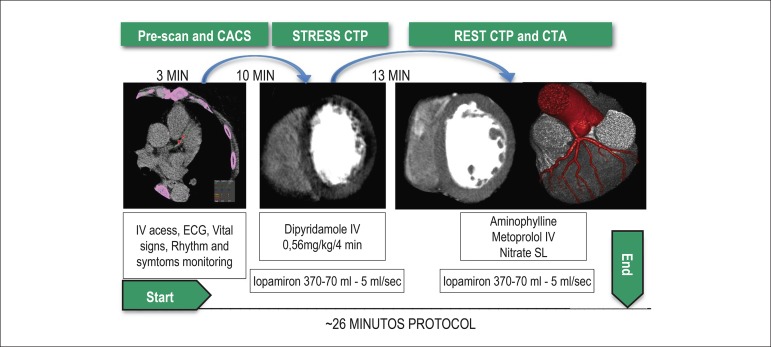
Stress Perfusion LBBB CT protocol. CACS: Calcium score; ECG: Electrocardiogram; Stress CTP: Stress myocardial perfusion; Rest CTP: Rest myocardial perfusion; CTA: Coronary angiography; IV: Intravenous; SL: Sublingual.

The study ran from February to December 2011. In this time, 3709 patients underwent stress myocardial perfusion scintigraphy. Of those patients, 87 patients who had LBBB and were under investigation for CAD, underwent stress myocardial perfusion scintigraphy with vasodilators (Figure 1). After reviewing the eligibility criteria, 30 patients were selected to undergo a CT study, which included three steps: Calcium score, myocardial stress perfusion scan with dipyridamole, and myocardial perfusion/coronary angiography at rest (Figure 2). These patients had undergone SPECT within the previous 2 months and were referred for invasive coronary angiography within 60 days of the cardiac CT.

All patients were instructed to descontinue beta-blockers for 48 h before the tests, and to stop caffeine and xanthines from their diet for 24 h prior to pharmacological stress for either the CT and SPECT tests. They were asked to fast for 6 h immediately before the scan.

### Pre-scan & Calcium score

A CT protocol was followed with the patient attached to electrocardiographic and blood pressure monitoring. It began with a CT scanogram of the chest. A Coronary Artery Calcium Score (CACS) scan was conducted from the carina to the bottom of the heart silhouette.

### Stress myocardial perfusion CT

The cardiac field of view (FOV) in the craniocaudal direction was determined on the basis of CACS. The 320-row scanner uses a collimation of 320 × 0.5-mm detector row and a gantry rotation of 350 ms, providing up to 16 cm of z-axis coverage in a single tube rotation, enough to capture the entire heart.

The stress scan was initiated 2 min after the end of an intravenous dipyridamole infusion (0.56 mg/kg), which was given through the right antecubital vein for 4 min. Real-time bolus tracking was performed, adjusting a region of interest (ROI) to a threshold of 210 HU (Hounsfield Unit) in the descending aorta. CTP acquisition was always performed within one heartbeat, including systolic and diastolic phases (40-80% R-R interval), after an infusion of 70 mL of intravenous contrast medium Iopamidol (Iopamiron 370 mg/mL; Bayer Schering Pharma, Berlin, Germany) at a rate of 5 ml/s, followed by 30 mL of a saline flush. The tube voltage (kV) and tube current (mA) were pre-determined according to the patient’s BMI (Table 1). During dipyridamole infusion symptoms, blood pressure and electrocardiographic parameters were continuously monitored.

**Table 1:** CT protocol acquisition parameters and results

Modality	Gating	kV			mA	
		< 29 Kg/m^2^		> 29 Kg/m^2^	< 29 Kg/m^2^	> 29 Kg/m^2^
CACS	Prospective	120		120	150	300
Stress CTP	Prospective	100		120	580	450-580
CTA and rest CTP	Prospective*	100		120	580	450-580

* One retrospective exam was made due to the tachycardia at rest.

**Table:** 

**Effective radiation exposure (mSv)**	**Dados**
Total Radiation dose of CT protocol	9.3 ± 4.6
CACS scan	0.8 ± 0.3
Stress scan	3.8 ± 3
Rest scan	3.7 ± 2
SPECT*	14.6 ±4.4
Other parameters	
Total Contrast material dose, (ml)	131 ± 10.3
CTA image quality (subjective analysis)	1.8 ± 0.9
**Heart rate (beats/min)**	
Basal heart rate on CT room	71.1 ± 11.6
Stress scan heart rate	87.3 ± 12.2
Increase heart rate with dipyridamole†	18%
Rest scan heart on CTA	62.7 ± 9

CACS: Calcium score; CTP: Computed tomography myocardial perfusion; kV: Tube voltage; mA: Tube current; mSv: MiliSievert; SPECT: Single photon emission computed tomography.

* Thallium-201 was used in two patients, with 99m TC-sestamibi being used in the remaining 28 patients.

† Mean value.

### Rest myocardial perfusion CT

After stress CTP acquisition, intravenous aminophylline (Aminophylline, University of São Paulo, São Paulo, Brazil) was administered (240 mg in 2 min) to reverse the vasodilator effect. Patients then received IV metoprolol (Seloken, AstraZeneca, São Paulo, Brazil), titrated to their blood pressure and other clinical criteria, with a maximum dose of 15 mg. This was continued until a target heart rate of < 65 beats/min was reached. A sublingual nitrate (2.5 mg Isordil, Sigma Pharma, São Paulo, Brazil) was used for inducing epicardial coronary artery vasodilation.

A rest perfusion/CTA scan was performed using prospective ECG gating, with a FOV that ensured the acquisition of the coronary arteries, keeping the same parameters as the stress scan (kV and mA) and the same contrast dose. An ROI in the descending aorta of 240 HU was programmed. To minimize radiation, the acquisition window was narrowed to target only diastolic phases of the cardiac cycle (60%-80% of R-R interval).

Whether at rest or during stress, myocardial perfusion was a static first-pass acquisition performed within a single phase of contrast injection. All parameters used for each of the three steps of the protocol are described in Table 1.

At the end of the protocol, the patients were re-examined and, if necessary, received 250-500 mL of saline solution intravenously in order to minimize the risk of contrast nephropathy and hypotension due to the vasodilators.

### SPECT

A pharmacological stress test was performed on all patients (adenosine or dipyridamole) using INFINIA, VENTRI I and II GE^®^ and CARDIO I and II Philips^®^ equipment. Half of the patients underwent each type of stress test. The standard institutional clinical protocol was followed, under which, the patients were injected with 10 mCi of technetium-99 m-sestamibi at rest. Gated SPECT images were acquired with a dual-detector gamma camera, with 64 projections, each for 20 s, in a noncircular 180° orbit. Adenosine stress tests were performed with an intravenous adenosine infusion at 140 mcg/kg/min for 6 min. The tracer (30 mCi of technetium-99 m-sestamibi) was injected at the third minute of infusion. Dipyridamole stress tests were performed with intravenous dipyridamole (0,56 mg/kg for 4 min) and the tracer was injected after 2 min. After 30-60 min, gated SPECT images were obtained.

The short axis, horizontal long axis, and vertical long axis were used to read the images. The method to reproduce the images obtained was interactive reconstruction. A pre-reconstruction filter was also used, with the aim of smoothing images and eliminating high-frequency noise.

### Analysis of CT & SPECT images and QCA

Two blinded observers independently analyzed the images. They were both physicians with over 4 years’ experience of interpreting CTA. Analysis of CTP and CTA were performed separately. Whenever they disagreed, a consensus had to be reached. None of the observers had received any clinical information or been made aware of the results of any other tests.

All analyses (CTP and CTA) were performed on a workstation (Vitrea FX, Vital Images, Minnetonka, MN, USA) using a visual and semi-quantitative approach and compared with QCA as the reference method with a reduction in the luminal diameter of 70% or more being considered significant.

Two blinded observers independently analyzed the SPECT images. They were both physicians with over 10 years’ experience interpreting SPECT. When there was a disagreement in the SPECT images between these two nuclear medicine physicians, a third reader helped them to reach a consensus. Similar to CT, the images of SPECT were analyzed using a visual and semi-quantitative approach.

Data analysis was performed to compare the accuracy of myocardium CTP and SPECT and to compare it against the gold standard of QCA.

The American Heart Association 17-segment model was used to identify perfusion defects. When comparing perfusion data (CTP vs. SPECT) with coronary anatomical data derived from CTA/QCA, we used the American College of Cardiology/American Heart Association recommendations to consolidate the segmental data into three territories. This is known as per-territory analysis^13^.

CTP data was evaluated on a true short axis using two and four chamber views, with multiplanar reformatted images that were an average of 8 mm thick. The appropriate window and level were also used (350W/150L)^9^. Initial evaluation of perfusion defects started in the diastolic phase. To avoid potential artifacts, readers used systolic phases to confirm the perfusion defect. A true perfusion defect was defined as subendocardial hypoenhancement encompassing > 25% of the transmural extent, which was present in different phases of the cardiac cycle and within a specific coronary territory.

CTA stenosis was graded as 0%-25% (minimal), 25%-49% (mild), 50%-69% (moderate), and ≥ 70% (severe)^13^. Coronary territories were classified by the highest degree of stenosis within their segments. All coronary segments were included in our analysis. Image data to evaluate stress myocardial perfusion defects was not used to analyze coronary anatomy. In addition, for each CTA image, a subjective measure of quality was obtained. These ranged from 1 to 5: 1 = excellent, 2 = good, 3 = moderate, 4 = poor, and 5 = blurred/non-diagnostic images.

We did not exclude any patients based on SPECT results and neither did we discard any coronary segment (n = 540) based on its diameter, importance, or due to coronary calcium on CTA analyses. No patient was excluded based on CTA or CTP image quality or other artifacts.

The additional value of CTP on CTA alone was calculated using QCA as a standard reference. The anatomical data (stenosis by CTA) was the decisive criteria for the final definition of the combination (negative or positive when stenosis was mild or severe, respectively). Therefore, when the patient had a mild stenosis (< 50%), the final combination with CTP was negative. When stenosis was severe (≥ 70%), the final combination was positive, regardless of the CTP results. But when stenosis was moderate (50%-69%), combined evaluation was considered positive or negative according to perfusion data (CTP)^14^. No reclassification of CTA stenosis was performed after the information of combined CTA and CTP.

QCA was performed using a semi-automated detection system (QCA for research 2.0.1, Pie Medical Imaging, Maastricht, Netherlands), by a biomedical scientist with training and over five years’ experience in QCA. They were blinded to the CTA, CTP, or SPECT results. It was performed in all patients who underwent invasive coronary angiography, including all coronary arteries with any degree of visual stenosis. In order to standardize coronary anatomy (CTA and QCA) analyses, an 18-segment coronary model was used^15^.

### Statistical analysis

Statistical analysis was performed using STATA 10.0 (STATA Corp, College Station, TX, USA). Continuous variables were expressed as mean ± SD, whereas categorical variables were expressed as percentages. Association between the methods was evaluated using sensitivity, specificity, accuracy, and predictive values. A kappa analysis was performed to evaluate agreement between CTP, CTA, SPECT, and QCA.

The association between categorical variables and the outcome of QCA ≥ 70% was assessed using a chi-square or Fisher exact test. The area under the receiver operating characteristic curve (AUC) was calculated and described with a 95% confidence interval (CI). *P* values < 0.05 were considered statistically significant.

## Results

Patient characteristics are summarized in Table 2. Of the 30 patients with LBBB, the mean age was 62 ± 10 years (60% were women, 30% were obese (BMI ≥ 30 Kg/m^2^), 10% were current smokers, and 42% were diabetics). Dyspnea and chest pain (60%) were the most frequent symptoms.

**Table 2:** Baseline characteristics of the 30 study patients

**Demographic data and Risc factor**	**Valores**
Age (years), mean ± SD	62 ± 10
White Ethnicity, n (%)	19 (63%)
Woman, n (%)	18 (60%)
Hypertension, n (%)	21 (71%)
Smoker, n (%)	3 (10%)
Diabetes, n (%)	14 (46%)
Dyslipidemia, n (%)	28 (93%)
Familiar history of IHD, n (%)	12 (40%)
Overweight (BMI > 25 kg/m^2^), n (%)	17 (56%)
Obesity (BMI > 30 kg/m^2^), n (%)	9 (30%)
Dyspneia, n (%)	18 (60%)
Chest pain, n (%)	18 (60%)
Ejection fraction, mean ± SD	42.4 ± 17
**Biomaker or lipid level (mg/dl)**	
Total cholesterol, mean ± SD	192 ± 57
HDL cholesterol, mean ± SD	45 ± 10
LDL cholesterol, mean ± SD	119 ± 41
Serum tryglyceride, mean ± SD	135 ± 95

IHD: Ischemic heart disease; BMI: Body mass index; SD: Standard deviation.

When this population was evaluated using the Framingham risk score, ten patients were intermediate risk and four were high risk. The average risk was intermediate in men and low in women (13% and 7% 10 year risk, respectively).

All 30 patients completed the CT protocol with a mean total radiation dose^16^ of 9.3 ± 4.6 mSv (Table 1). The only three cases of adverse events were mild nausea, most likely due to the contrast infusion. One patient who underwent invasive coronary angiography showed a local adverse effect (a mild hematoma at the site of puncture). No other minor or major events were observed that were related to the research protocol.

Invasive coronary angiography was performed in all patients. Analyses were made per-patient and per-territory for CTP, CTA, and SPECT, using QCA as the reference standard (considering significant coronary stenosis ≥ 70%; Table 3).

**Table 3:** Diagnostic accuracy of CT protocol and SPECT in per-patient and per-territory analysis

		**Per-Patient Analysis ‡**				**Per-Territory Analysis ‡**					
		**CTP**	**CTA**	SPECT*	**CTP + CTA**	**CTP**	**CTA**		**SPECT** †	**CTP + CTA**	
Accuracy		83%	90%	63%	90%	79%	91%		69%	89%	
Sensitivity		86%	86%	97%	93%	63%	85%		44%	85%	
Specificity		81%	94%	32%	87%	86%	94%		79%	90%	
PPV		80%	92%	56%	87%	65%	85%		48%	79%	
NPV		87%	88%	92%	93%	84%	94%		77%	93%	
**A**						**B**					
**CTP**		**QCA ≥ 70%**				**CTP**	**QCA ≥ 70%**				
		**-**	**+**	**Total**			**-**		**+**	**Total**	
-		13	2	15		-	54		10	64	
+		3	12	15		+	9		17	26	
Total		16	14	30		Total	63		27	90	
p = 0.001						p < 0.001					
**A**						**B**					
**SPECT**		**QCA ≥ 70%**				**SPECT**	**QCA ≥ 70%**				
		**-**	**+**	**Total**			**-**		**+**	**Total**	
-		5	0	0		-	50		15	65	
+		11	14	25		+	13		12	25	
Total		16	14	30		Total	63		27	90	
p = 0.045						p < 0.021					

Data of 30 patients and 90 territories using QCA as reference standard (considering coronary stenosis ≥ 70%). PPV: Positive predictive value. NPV: Negative predictive value.

* In SPECT per-patient analysis, the presence of fixed defect or/and ischemia was used.

† In SPECT per-territory analysis only ischemia was used.

‡ p < 0.05.

A -Per-patient analysis. B- Per-territory analysis.

**Table:** 

**C**	**Per-Patient Analysis**			**Per-Territory Analysis**		
	**CTP** §	**CTA**	**SPECT** §	**CTP**	**CTA**	**SPECT** §
Accuracy	70%	87%	63%	71%	84%	59%
Sensitivity	67%	89%	89%	50%	73%	36%
Specificity	75%	83%	25%	90%	94%	79%
PPV	80%	89%	64%	81%	91%	60%
NPV	60%	83%	60%	67%	80%	58%

C Data of 30 patients and 90 territories using QCA as reference standard (considering coronary stenosis > 50%).

§ p > 0.05. The others analysis p < 0.05.

**Figure 3:**
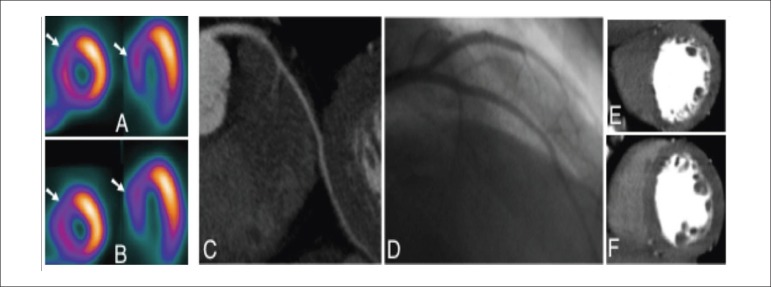
Patient number 30, a SPECT false-positive caused by LBBB. (A) Stress and (B) rest SPECT showing a fixed defect in the anteroseptal wall (white arrows). CTA on curved multiplanar reformatted image (C) and invasive coronary angiography (D) show a normal left anterior descending coronary artery. Normal Stress (E) and Rest (F) CTP.

**Figure 4:**
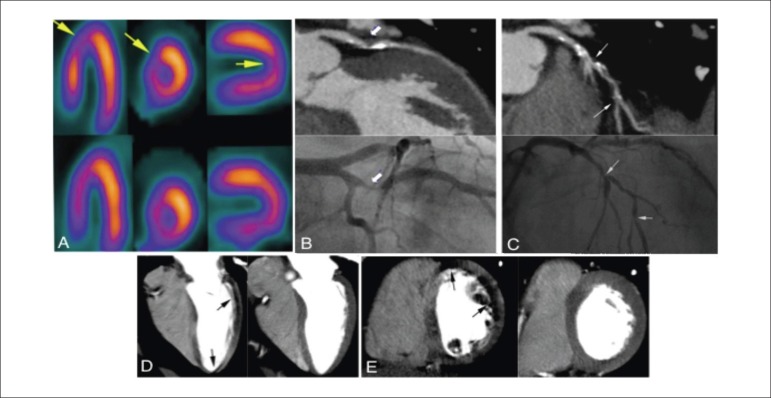
Patient number 12, a better correlation between CTP/CTA and QCA than SPECT. (A) Mild ischemia in the apical wall in SPECT demonstrated by yellow arrows at stress and normal at rest. (B) Severe stenosis in the proximal obtuse marginal showed on curved multiplanar reformatted CTA image (above) and invasive coronary angiography (below). (C) Severe stenosis in the left anterior descending at proximal and midportion shown on curved multiplanar reformatted CTA image (above) and invasive coronary angiography (below). (D) 4-chamber CTP demonstrating ischemia in the lateral and apical walls (black arrows). (E) Short-axis CTP showing ischemia in the anterior and lateral walls (black arrow).

The sensitivity, specificity, positive, and negative predictive values and accuracy were 86%, 81%, 80%, 87%, and 83% respectively for per-patient analysis and 63%, 86%, 65%, 84%, and 79%, respectively for per-territory analysis (n = 90 territories) in relation to CTP (p < 0.001; Figures 3 and 4).

Regarding SPECT results, in per-patient analysis considering ischemia and/or fixed defects, the sensitivity, specificity, positive and negative predictive values and accuracy were 97%, 32%, 56%, 92% and 63% respectively (p = 0.045). In per-territory analysis, relating only patients with ischemia and excluding those with fixed defects on SPECT, the values were 44%, 79%, 48%, 77% and 69%, respectively (p = 0.021) evaluated by QCA (stenosis ≥70%) as reference standard.

In table 3, the results are also given for a QCA stenosis of > 50%. Comparing the results (QCA ≥ 70% versus > 50%), it is clear that for both methods, CTP and SPECT, the accuracy was better with a QCA ≥ 70%.

Almost half of the patients (14/30) had a SPECT examination that was influenced by a septal defect that was due to LBBB. When QCA was conducted, five of these were found to be true positives and nine were false positives. From this group of fourteen patients, seven had normal CTP and QCA.

When per-patient analysis was carried out, the interobserver agreement (kappa) was considered moderate for CTP (k = 0.53; p < 0.05) and SPECT (k = 0.41 p < 0.05); however, the figures were slightly better for CTP (23/30) than SPECT (21/30).

The median CACS was 212. From the 30 patients, four had a CACS of zero; eight had a CACS between 0 and 100; ten had a CACS between 100 and 400; six had a CACS between 400 and 1000 and two had a CACS, which was more than 1000.

The CACS mean Agatston score was 512 (244-814), which is on the 93^rd^ percentile (91-98, p < 0.001) for the group who had a coronary stenosis of ≥ 70%, on QCA. This was closely related to the CAD burden according to gender, race, and age^17^.

### Incremental value of CTA on CTP

There were 5 patients with moderate stenosis, corresponding to 6 coronary segments on CTA. Addition of CTA to CTP significantly improved sensitivity (to 85%), whilst keeping the high specificity of 90% (p < 0.0001). The overall accuracy for detection of functionally significant CAD was 79% for CTP, 70% for SPECT, and 89% for the integrated protocol (CTA + CTP) in per-territory analysis (p < 0.0001).

Comparison of AUCs in per-patient and per-territory analysis, looking at SPECT, CTP, CTA and the combination CTA and CTP with QCA as the gold standard (stenosis ≥ 70%) is shown in graphic 1.

**Graphic 1:**
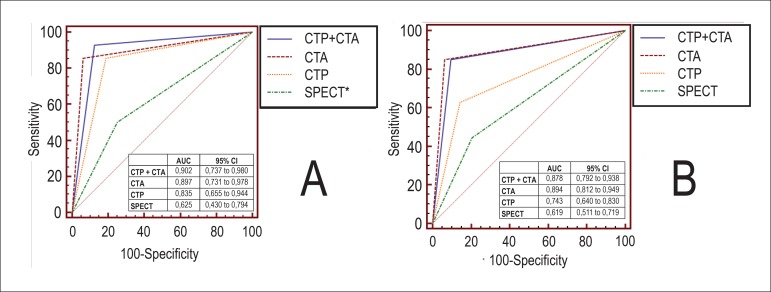
AUC comparing CTA, CTP, SPECT, and combination CTP + CTA with QCA as reference.

A -Per-patient analysis. B -Per-territory analysis. All analysis have p < 0.05, except for SPECT per-patient analysis (p = NS. * p = 0.16).

In per-patient analysis, CTA and CTP had the highest accuracy (AUC of ROC curve = 0.90; p < 0.0001). CTP AUC was 0.83 p < 0.0001. Comparisons between CTP vs. SPECT and CTP + CTA vs. SPECT showed the superiority of CT methods over SPECT (AUC differences of 0.210 p = 0.038 and 0.277 p = 0.017, respectively).

In per-territory analysis, there was also a significant improvement in accuracy when comparing the integrated protocol CTA + CTP versus SPECT, showing a difference between areas of 0.259 (p = 0.0004).

### Results of CTA vs. QCA

Results of CTA compared with QCA on per-patient and per-territory analysis (using as a threshold a coronary stenosis ≥ 70%) are displayed in table 3. The results showed excellent accuracy with a sensitivity value of 86%, specificity of 94%, positive predictive value of 92% and negative predictive value of 88% (p < 0.05).

We used intravenous metoprolol (average dose 15 mg) to reduce the mean heart rate to 63 beats/min (a reduction of almost 10 beats/min) to enable a good quality CTA to be performed after the stress scan.

Of the patients referred for invasive coronary angiography, nine (30%) had normal coronary arteries, three (10%) had minimal or mild coronary artery disease (stenosis < 50%), 14 (46%) had stenosis > 70%, four (13%) had single-vessel coronary artery disease (stenosis > 50%), two (7%) had two-vessel coronary artery disease, and 12 (40%) had triple-vessel coronary artery disease.

## Discussion

The difficulty in evaluating ischemia in patients with LBBB represents an important area for study, especially given the limitations of current techniques. To the best of our knowledge, this was the first study looking at patients with LBBB and using the latest generation 320-row detector CT scanner to analyze the accuracy of CTP using a combination of coronary angiography and myocardial stress perfusion with dipyridamole. This study also showed that a feasible and comprehensive protocol was able to evaluate the CAD burden using CACS, myocardial perfusion imaging during stress and at rest, and a measure of coronary anatomy with good diagnostic accuracy. There was a low rate of adverse events, an acceptable dose of radiation and admissible duration of exam.

A previous study^3^ showed that CTA could be an alternative to stress tests in screening patients with LBBB. The accuracy of CTA can however be decreased by arrhythmia, motion artifacts, or excessive calcifications and the degree of coronary obstruction measured by CTA or conventional angiography remains a poor predictor of reversible ischemia due to atherosclerosis^12,18^.

A combined evaluation of CTP on CTA during pharmacologic stress under a single examination has recently been described and validated^7-11^. Besides the information about myocardial ischemia, the addition of stress myocardial CTP can also give additional data to some non-assessable segments, improving diagnostic accuracy^19^.

Myocardial ischemia is an important factor that determines clinical outcomes^20^ and benefit from revascularization^21^. The stress CTP has been evaluated in numerous studies^22^ and has proven to be an alternative stress test.

Despite the fact that CTP is not a dynamic test, there is a great opportunity to visualize the differences of x-ray attenuation between ischemic and remote myocardium. A potential advantage of CTP over SPECT is the ability to acquire high-resolution isotropic 3D images that allow simultaneous coronary anatomy and myocardial perfusion analysis. This may be of particular interest for decision-making regarding revascularization.

In a recent review evaluating 14 studies^22^, the sensitivity of CTP ranged from 79% to 97% with specificity from 72% to 98% depending on the scanner type, reference stenosis standard, studied population, and whether analysis is per patient or territory. This agrees with our results that demonstrated a sensitivity of 79% and specificity of 86% for CTP, when using per-territory analysis. The prevalence of obstructive atherosclerosis in this study was 60%, which was similar to previous studies; 59% in the study by George et al^12^ and 69% in the study by Cury et al^9^. In our protocol, the addition of CTP to CTA increases CT global accuracy for functionally significant CAD in patients with LBBB, mainly because of a significant increase of sensitivity whilst keeping a high specificity. Thus, a 320-row detector CT scanner with an anatomical and functional integrated protocol may be effective for the detection of functionally significant CAD in patients with LBBB.

This study could evaluate the presence, extension, and severity of CAD. The total radiation exposure of 9.3 ± 4.6 mSv in our CT protocol was lower than SPECT (14.6 ± 4.4 mSv), which provides only an assessment of perfusion, not of the coronary anatomy.

Compared to recent related studies^11,22^, this study used a low rate of ionizing radiation. This could be explained by some factors: Physical parameters (kV and mA) were adapted to the BMI of the patient and kept as low as reasonably possible. Prospective acquisition limiting radiation was applied only to a short interval of the electrocardiogram. The field of view was limited to the heart and the perfusion images were acquired using only one beat for each phase. AIDR technology (Adaptive Dose Reduction Interactive) was not available at the time of this study, but if used would further reduce the radiation dose.

It is theoretically possible that inducing tachycardia during the stress CTP could create artifacts, which could mimic or mask a perfusion deficit. There is no evidence that this happened. There was no verifiable increase in interference in the septal and apical walls caused by high heart rates.

The interobserver concordance for CTP was moderate and this could be explained by the fact that CTP is a new technique and involves a certain degree of uncertainty and a learning curve.

In this study, we selected stenosis ≥ 70% instead of > 50% as anatomical reference for QCA. The literature shows the use of both cut-offs to compare CT perfusion stress tests^9,11^. Although studies^18,23^ reveal that there is functional repercussion with coronary stenosis from 40%, a coronary flow reserve was found to vary widely among patients with stenosis of 50 to 70 percent. The mean reason to select ≥ 70% as the anatomical reference was the potential to have more definite results for CTP and SPECT when compared to QCA. For the intermediate results on CTA (50-69%), we were guided by the literature to choose the appropriate functional test to make decisions on revascularization. SPECT is often performed to detect CAD in patients with LBBB. However, stress scintigraphy is not specific due to the frequent occurrence of septal, anterior, and apical defects in the absence of CAD. Specificity has been reported to be low due to false-positive septal perfusion abnormalities, and it had already been shown that specificity could be improved using a dipyridamole stress test. Recently, Fovino et al. reported that the presence of myocardial ischemia on SPECT was the only predictor of events in patients with LBBB who had a low or intermediate cardiac risk and were followed for 32 ± 18 months^24^.

In our analysis, SPECT demonstrated a high negative predictive value in per-patient analysis (92%). Thus, patients with LBBB and normal scintigraphy would not need further investigation or invasive strategy; on the other hand, patients who have an abnormal result may need additional cardiac evaluation for appropriate management. These findings are in agreement with the literature and confirm the high negative predictive value of SPECT with pharmacological stress in patients with LBBB^25^.

### Limitations

This was a single-center study with a small number of patients, so our findings need confirmation from larger multicenter studies.

The majority of patients with obstructive CAD on invasive angiography had triple vessel disease. These patients were followed at the cardiology clinic of a tertiary hospital and referred for evaluation with SPECT due to a high risk of CAD and a high prevalence of cardiac risk factors such as diabetes and hypertension. Our data is therefore most useful for populations with high levels of CAD.

QCA is the most currently used anatomic reference method. Due to financial issues, fractional flow reserve was not performed in our group of patients.

## Conclusion

We demonstrated that the combination of anatomical and functional information in a single CT examination is feasible and has good accuracy for the detection of obstructive CAD in patients with LBBB. The results of the study suggest that stress perfusion CT, performed with a 320-row detector CT scanner, can be an alternative strategy to patients with LBBB who need evaluation for myocardial ischemia.
